# Cubic Silver Nanoparticles Fixed on TiO_2_ Nanotubes as Simple and Efficient Substrates for Surface Enhanced Raman Scattering

**DOI:** 10.3390/ma12203373

**Published:** 2019-10-16

**Authors:** Robert Ambroziak, Marcin Hołdyński, Tomasz Płociński, Marcin Pisarek, Andrzej Kudelski

**Affiliations:** 1 Faculty of Chemistry, University of Warsaw, Pasteur Str. 1, 02-093 Warsaw, Poland; rambroziak@chem.uw.edu.pl (R.A.); akudel@chem.uw.edu.pl (A.K.); 2 Institute of Physical Chemistry Polish Academy of Sciences, Kasprzaka Str. 44/52, 01-224 Warsaw, Poland; mholdynski@ichf.edu.pl; 3 Faculty of Materials Science and Engineering, Warsaw University of Technology, Woloska 141, 02-507 Warsaw, Poland; tomasz.plocinski@pw.edu.pl

**Keywords:** plasmonic materials, SERS (Surface Enhanced Raman Spectroscopy), freestanding titanium oxide nanotubes (TiO_2_ NTs), cubic silver nanoparticles (Ag CNPs), XPS (X-ray photoelectron spectroscopy)

## Abstract

In this work we show that ordered freestanding titanium oxide nanotubes (TiO_2_ NT) may be used as substrates for the simple and efficient immobilization of anisotropic plasmonic nanoparticles. This is important because anisotropic plasmonic nanostructures usually give greater spectral enhancement than spherical nanoparticles. The size of the pores in a layer of titanium oxide nanotubes can be easily fitted to the size of many silver plasmonic nanoparticles highly active in SERS (surface-enhanced Raman scattering) spectroscopy (for example, silver nanocubes with an edge length of ca. 45 nm), and hence, the plasmonic nanoparticles deposited can be strongly anchored in such a titanium oxide substrate. The tubular morphology of the TiO_2_ substrate used allows a specific arrangement of the silver plasmonic nanoparticles that may create many so-called SERS hot spots. The SERS activity of a layer of cubic Ag nanoparticles (AgCNPs) deposited on a tubular TiO_2_ substrate (AgCNPs@TiO_2_ NT) is about eight times higher than that of the standard electrochemically nanostructured surface of a silver electrode (produced by oxidation reduction cycling). Furthermore, a super hydrophilic character of the TiO_2_ nanotubes surface allows for a uniform distribution of AgCNPs, which are deposited from an aqueous suspension. The new AgCNPs@TiO_2_ NT hybrid layer ensures a good reproducibility of SERS measurements and exhibits a higher temporal stability of the achievable total SERS enhancement factor—one that is far better than standard SERS silver substrates. To characterize the morphology and chemical composition of such evidently improved SERS platforms thus received, we applied microscopic techniques (SEM, and scanning transmission electron microscopy (STEM)) and surface analytical techniques (Auger electron spectroscopy (AES) and X-ray photoelectron spectroscopy (XPS)).

## 1. Introduction

Raman scattering is an optical effect with efficiency being usually very low. This is due to the fact that Raman scattering is weaker than Rayleigh scattering, by about 10^−6^–10^−7^ times. A typical cross-section for Raman scattering is ca. 10^−29^ cm^2^ per molecule, whereas typical cross-sections for absorption in ultraviolet and infrared are ca. 10^−18^ and 10^−21^ cm^2^ per molecule, respectively [1]. Therefore, for many decades Raman spectroscopy was not considered as a useful analytical tool. With the discovery of the surface-enhanced Raman scattering (SERS) phenomenon in the 1970s of the last century, it allowed for a huge enhancement of recorded Raman signals for molecules (pyridine) adsorbed on silver with a roughened surface [2,3,4]. When electromagnetic radiation interacts with a nanostructured surface of a metal with a negative real and small positive imaginary dielectric constant (e.g., silver when visible radiation is used), these induce a collective oscillation of surface conduction electrons, called surface plasmons, that leads to enhanced electromagnetic fields at some locations near the surface of the illuminated plasmonic nanostructure [1,5]. The local increase in the intensity of the electric field near illuminated plasmonic nanostructures may cause a significant increase in the efficiency of Raman scattering (for Raman bands with a small Raman shift, this increase is roughly proportional to the fourth power of the field enhancement [1]). In some cases, the nanoresonator-induced increase in the efficiency of the generation of the Raman signal is so large that the Raman scattering cross-section can even be increased up to, e.g., 2 × 10^−14^ cm^2^ per molecule [6] (i.e., about 15 orders of magnitude in comparison with normal Raman scattering) which makes it possible to observe the Raman spectra of even a single molecule [6,7,8]. SERS spectroscopy is one of the most sensitive analytical tools available, and is widely used in medicine [9,10,11], forensic science [12], the food industry [13,14,15] and analytical chemistry [16,17,18]. The sensitivity of SERS measurements is strongly dependent on the activity of plasmonic material used as the SERS substrate, and so the fabrication of such plasmonic nanomaterials is an important and rapidly-developing branch of nanotechnology. The quality of the SERS spectra measured strongly depends on the activity and reproducibility of the plasmonic material used as the SERS substrate. Therefore, many groups are working on developing new methods of fabricating SERS substrates. Materials for SERS measurements may be obtained, for example, by nanostructuring the surface of a plasmonic metal, evaporating a plasmonic metal on the nanostructured surface of a non-plasmonic material, or depositing plasmonic nanostructures on the surface of plasmonic or non-plasmonic materials. This last approach, currently being widely investigated, makes it possible to easily create SERS substrates with strongly anisotropic plasmonic nanostructures and many sharp apexes and edges (such plasmonic structures are especially useful for SERS measurements because, for illuminated plasmonic nanoparticles, the highest electromagnetic field enhancement is observed at their sharp apexes and edges [19,20]). What is important here, for anisotropic plasmonic metal nanoparticles (NPs) distribution of intensity gain of the electromagnetic field around NPs is strongly heterogeneous, what was confirmed by DDA method (discrete dipole approximation). The DDA method refers to the exact solution of the problem of light scattering on non-spherical and heterogeneous particles and on periodic particle systems [21] based on Mie theory [22], which states that small spherical nanocrystals should exhibit only a single surface plasmon resonance (SPR), whereas anisotropic particles would have two or more SPR, depending on their shape [23]. 

In this work, we show that ordered, freestanding titanium oxide nanotubes can be used as efficient and mechanically/chemically stable substrates for the immobilization of plasmonic nanoparticles. Although freestanding titanium oxide nanotubes adorned with spherical plasmonic metals (Ag, Au or Cu) have already been used as SERS substrates [24,25,26], highly anisotropic plasmonic nanostructures have not been successfully deposited on such nanotubular arrays, yet. This substrate due to the appropriate surface geometry and physicochemical properties, guarantee a uniform distribution of anisotropic plasmonic nanoparticles on the surface. The size of the tube in a layer of titanium oxide nanotubes can be easily fitted to the size of the deposited plasmonic nanoparticles; during the electrochemical synthesis of titanium dioxide nanotubes, it is possible to control their size and the thickness of their walls through anodic voltage and electrolyte composition [27,28,29,30]. These factors affect the specific surface morphology of such kind of materials [31,32]. That is why, the deposited plasmonic nanoparticles can fit well in the slits and pores created, and can therefore, be strongly anchored in the nanotubular TiO_2_ substrate. The tubular morphology of the TiO_2_ substrate allows a specific arrangement of silver plasmonic nanoparticles that creates many so-called SERS hot spots (mainly narrow slits between the plasmonic nanostructures). Therefore, the surface roughness, size and shape of the nanoparticles are used to decorate TiO_2_ nanotubes influence on the value of enhancement Raman scattering. Achieving the maximum signal during SERS measurements requires optimization of the mentioned parameters [33]. Thus, conventional materials such as rough surfaces of noble metal electrodes obtained in the oxidation reduction cycle, and colloid nanoparticles are increasingly less important due to the development of nanotechnology. However, interesting issues at the moment concern the possibility to obtain plasmonic nanoparticles immobilized on larger nanoparticles that do not give the SERS spectrum (such as silica nanoparticles) [34], or on nanostructural substrates such as graphene oxide [35], and also to directly making aggregates in solution [36,37] on a macroscopic substrate using molecules that are linkers [37] or even using microfluidic systems [37]. The new solutions for the design and production of SERS active substrates appear all the time in the literature, they lead to the development of simple ways that can provide adequate enhancement of the measured SERS signal and the repeatability of platform production processes. In the present study, for the first time we compare the SERS results of cubic silver nanoparticles fixed on TiO_2_ nanotubes with reference to the standard silver surface obtained on the basis of oxidation–reduction cycling procedure (standard SERS substrate) [4]. 

## 2. Materials and Methods

### 2.1. Materials

Trisodium citrate dihydrate, silver nitrate, potassium chloride, ethylene glycol and acetone were purchased from POCH S.A. (Gliwice, Poland). Sodium sulphide was acquired from Sigma-Aldrich (St. Louis, MO, USA). Polyvinylpyrrolidone (PVP) with an average molar mass of ca. 4 × 10^4^ g mol^−1^ was purchased from Fluka (Seelze, Germany). Glycerin and ammonium fluoride were purchased from Chempur (Piekary Slaskie, Poland). Pyridine was purchased from Ubichem (Eastleigh, UK). All of the chemicals were used without further purification or treatment. The water was purified by a Millipore Milli-Q system and had a resistivity of ca. 18 MΩ cm^−1^. The silver used as a material for electrodes was purchased from the Polish Mint (Warsaw, Poland). Titanium foil of 0.25 mm thick and 99, 5% purity was purchased from Alfa Aesar (Kandel, Germany). It was cut into 1 cm^2^ round plates. The plates were cleaned ultrasonically with acetone and ethanol, rinsed with deionized (DI) water, and dried in air.

### 2.2. Synthesis of Silver Cubic Nanoparticles by Skrabalak Method

**Figure 1:**
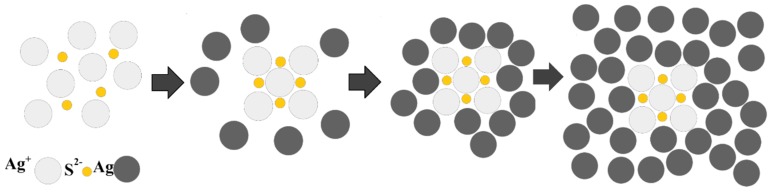
Scheme of synthesis of silver nanocubes.

All cubic silver nanoparticles described in this paper were synthesized using the modified method described by Skrabalak et al. [38]. The modification consisted in increasing the volume of the obtained solution and a slight change in the synthesis temperature. Briefly, 60 mL of glycol was poured into a three-necked flask equipped with a reflux condenser and magnetic stirrer. The solution was heated to 170 °C in one hour, and then 1 mL of a 3 mM Na_2_S solution and 15 mL of a 180 mM PVP (in respect to the monomer) solution were added. After stabilization of the temperature at 170 °C, 5 mL of a 280 mM solution of AgNO_3_ was added. All the solutions during the synthesis were prepared in glycol. After the addition of silver nitrate, the color of the reaction mixture changed from transparent through orange and red to dark green. After the last color change, the flask was placed in an ice bath to stop the reaction. The use of sulphides aimed at rapid nucleation on which a layer of reduced silver grows (see Figure 1). 

To transfer the nanoparticles from glycol to water, the solution was diluted with acetone 10 times and then centrifuged for 5 min. The supernatant was removed, water was added, and the mixture thus obtained was sonicated for 3 min. The centrifugation and suspension of the nanoparticles was then repeated twice. The mixture of Ag cubic nanoparticles (CNPs) suspended in water was then diluted 10 times as compared to the original solution. These procedures were intended not only to transfer nanoparticles to the water solution but also to remove most non-cubic nanoparticles of larger and smaller sizes than Ag nanoparticles (AgCNPs). When centrifugation was interrupted at the right moment, heavier cubic nanoparticles were deposited at the bottom, while nanoparticles of smaller mass that were spherical remained in the supernatant. Such prepared Ag CNPs nanoparticles were used to characterize them by scanning transmission electron microscopy (STEM) (FEI Nova NanoSEM 450, Brno, Czech Republic; Hitachi HD-2700, Naka, Japan), and UV-vis methods and to prepare appropriate SERS substrates.

### 2.3. Preparation of Titanium Oxide Nanotubes Substrates

Array 3646A and Motech LPS—303 power supplies were used for the electrochemical synthesis of TiO_2_ nanotubes (NT) onto Ti foil (0.25 mm thick) at a constant voltage of 25 V for 2 h in an optimized solution based on a glycerol and water (1:1 v/v) containing 0.8% wt. of NH_4_F. After anodic oxidation process of the Ti substrate, the samples were rinsed with water (24 h) and dried in air. Subsequently, thermal annealing in air was performed at 450 °C for 2 h to obtain the mechanically stable structure in anatase form [39].

### 2.4. Deposition of Silver Cubic Nanoparticles on Titanium Oxide Nanotubes

**Figure 2:**
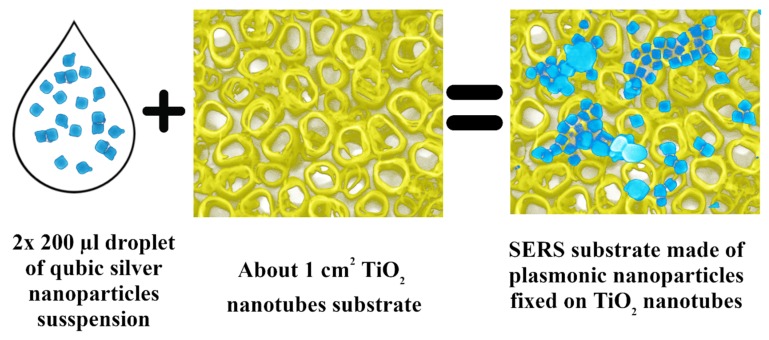
Scheme of preparation surface-enhanced Raman scattering (SERS) substrate based on titanium oxide nanotubes and cubic silver nanoparticles.

The 200 µL of an AgCNPs suspension was dropped onto the titanium oxide nanotubes (TiO_2_ NT) surface and allowed to dry overnight. This procedure was repeated once. The resulting substrates were used without any further preparation (see Figure 2).

### 2.5. Preparation of Electrochemically Nanostructured Silver

Reference SERS-active silver samples (Ag reference) were prepared by the electrochemical roughening of a pure Ag sheet, as in our previous work [33]. The roughening of the silver electrode was carried out in a conventional three-electrode cell with a large platinum sheet as a counter electrode and an Ag/AgCl (0.1 M KCl) electrode as a reference (all potentials are quoted versus this electrode). Five negative-positive oxidation reduction cycles were performed—from −300 to 300 mV at a sweep rate of 5 mV/s. The silver electrode was kept at −300 mV for 30 s and was removed at an open circuit. Finally, it was gently rinsed with water.

### 2.6. Characterization of Obtained Nanostructures

For the morphological characterization of the samples after their anodization, heat treatment and deposition of AgCNPs, scanning electron microscopy (SEM) and scanning transmission electron microscopy (STEM) examinations were carried out with an FEI Nova NanoSEM 450 instrument (FEI Nova NanoSEM 450, Brno, Czech Republic). SEM images were collected using the through lens detector (TLD) (FEI, Brno, Czech Republic) of secondary electrons at primary beam energy of 10 kV. The STEM studies were performed on samples deposited on a TEM grid (Quantifoil R2/2, 300 Cu mesh, ), using the bright field (BF) contrast mode of the detector (two segment planar solid-state p–n junction) attached under the grid holder, with high acceleration voltage of 30 kV. UV−Vis spectra were made using a Thermo Scientific Evolution 201 spectrophotometer (Waltham, MA, USA). The TEM observations at ultra high resolution were carried out using a Hitachi dedicated STEM 200kV equipped with a Cs corrector. The observations with bright field (BF) and high angle annular dark field (HAADF) detectors, as well as the convergent beam diffraction (CBD), were done to confirm the crystalline structure of the TiO_2_ phase.

### 2.7. SERS Measurements

SERS Raman spectra were measured using a Horiba Jobin-Yvon Labram HR800 spectrometer (Longjumeau, France) equipped with a Peltier-cooled CCD detector (1024 × 256 pixels), a 600 groove/mm holographic grating, and an Olympus BX40 microscope (Tokyo, Japan) with a long distance 50× objective. All Raman spectra were recorded using a diode pumped, frequency doubled Nd:YAG laser (532 nm). The 200 µL of 0.05 M pyridine in 0.1 M KCl was applied to the AgCNPs@TiO_2_ NT substrate, and the measurement itself was performed before the drop dried. For samples without pyridine, the spectra were collected without prior preparation of the substrate. For each substrate, 400 spectra were collected from a square surface with an edge length of 200 µm.

Substrate resistance tests were carried out for cleaning by rinsing with water. For this purpose, the substrate on which the above-described SERS measurements were made was rinsed several times with water from a wash bottle and then the spectra were recorded without pyridine in order to check whether it adsorbed on the surface. The above-described measurements with pyridine were then carried out. These activities were repeated to reach 14 consecutive cleaning cycles.

### 2.8. X-ray Photoelectron Spectroscopy (XPS) Measurements

The chemical composition and chemical state of the prepared SERS substrates were characterized by Auger electron spectroscopy (AES) and X-ray photoelectron spectroscopy (XPS). For this purpose, a Microlab 350 spectrometer (Thermo Electron) (East Grinstead, UK) was used. The XPS spectra were excited using Al_K__α_ (hν = 1486.6 eV, 300 W) radiation as a source. The survey and high resolution spectra were collected with the hemispherical analyzer at constant pass energies of 100 and 40 eV, respectively. The background was corrected using the Shirley model to obtain the XPS signal intensity. An asymmetric Gaussian/Lorentzian function at a constant ratio G/L = 0.3 was used for deconvolution procedure. The determined peak positions were corrected in relation to adventitious carbon C1s at 285.0 eV. Avantage Surface Chemical Analysis software (ver. 5.9904) was used for the data processing. The local Auger spectra were recorded at E = 10 kV, with the step of 1.0 eV.

## 3. Results and Discussion

### 3.1. Synthesis and Characterization of AgCNPs@TiO_2_ NT Hybrid Materials

#### Characterization of Cubic Silver Nanoparticles and Titanium Oxide Nanotubes

**Figure 3:**
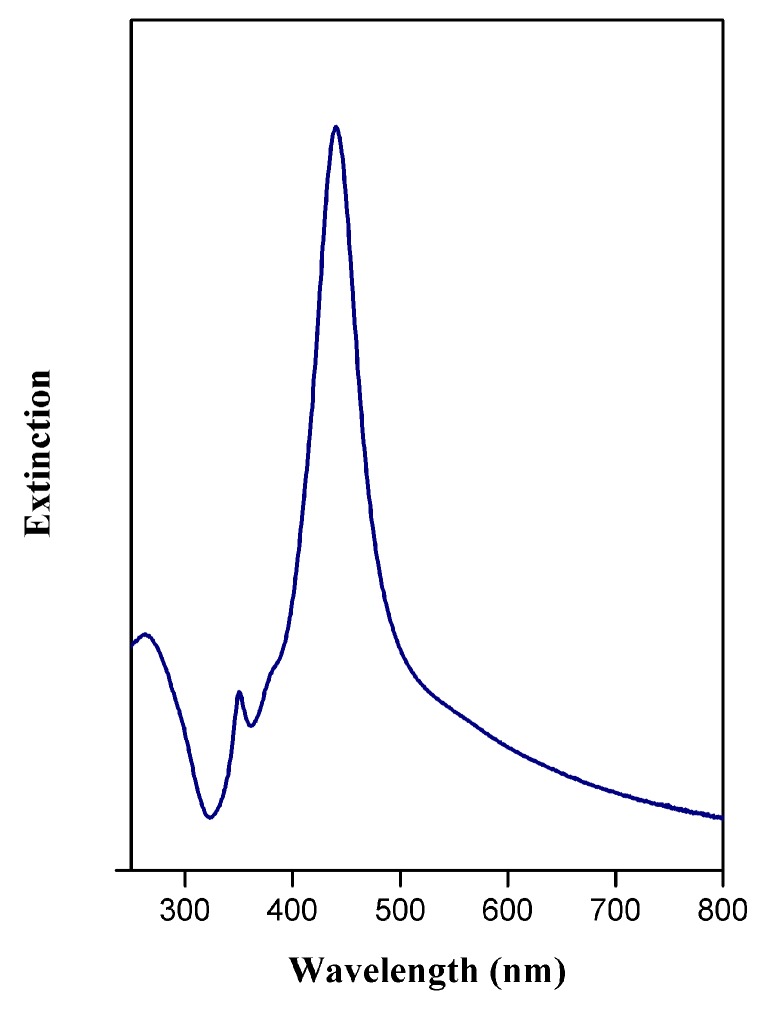
UV–Vis spectrum of cubic silver nanoparticles (AgCNPs).

**Figure 4:**
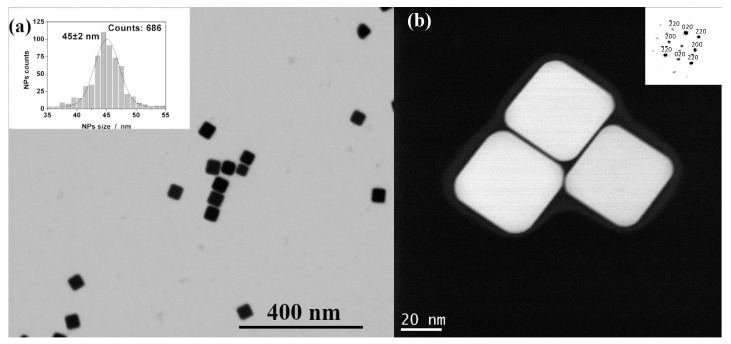
Scanning transmission electron microscopy (STEM) image of cubic silver nanoparticles deposited on silicon wafer (**a**) and TEM microscopic grid (**b**). The inserts show the histogram of Ag nanoparticles count vs. nanoparticle edge length (**a**) and the electron diffraction pattern (**b**).

After the synthesis of AgCNPs, UV-Vis spectra, to provide information about the optical properties of the prepared silver nanoparticles (see Figure 3) and STEM imaging (see Figure 4) to characterize their sizes and shapes, were performed. The recorded UV-Vis spectrum was typical for silver cubic nanoparticles [40], with the main surface plasmon resonance (SPR) extinction band at 440 nm. The regular cubic shape of the synthesized silver nanoparticles was confirmed by STEM measurements (see Figure 4). The average size of the nanoparticles was determined based in the image analysis. The histogram (nanoparticle count vs. nanoparticle edge length) presented in Figure 4a (insert) shows that the most probable particle edge length is 45 ± 2 nm. A careful inspection of the AgCNPs morphology revealed that the nanoparticles were coated with a thin film of PVP polymer (polyvinylpyrrolidone) and formed core-shell structures (see Figure 4b). The nanoparticles were permanently coated with PVP which cannot be removed during the applied purification process. PVP polymer was used in this study as a surface stabilizer, growth modifier, and nanoparticle dispersant during the formation of nanoparticles having a specific shape. The polycrystalline nature of the AgCNPs was confirmed by the diffraction pattern obtained from the bulk of the nanoparticles. The electron diffraction images obtained were assigned to the (001) plane of the face-centered cubic silver [41,42]. Such kind of plasmonic nanoparticles may effectively support surface plasmon resonance due to the formation of special sites called “hot spots” that amplify the SERS signal [43,44]. Cubic nanoparticles allowed us to control the plasmon resonance wavelengths from visible light to near IR, because anisotropy provides an additional degree of freedom when compared to spherical nanoparticles. Mainly, the strong LSPR (Localized Surface Plasmon Resonance)effect was localized at corners of AgCNPs [21,45]. 

**Figure 5:**
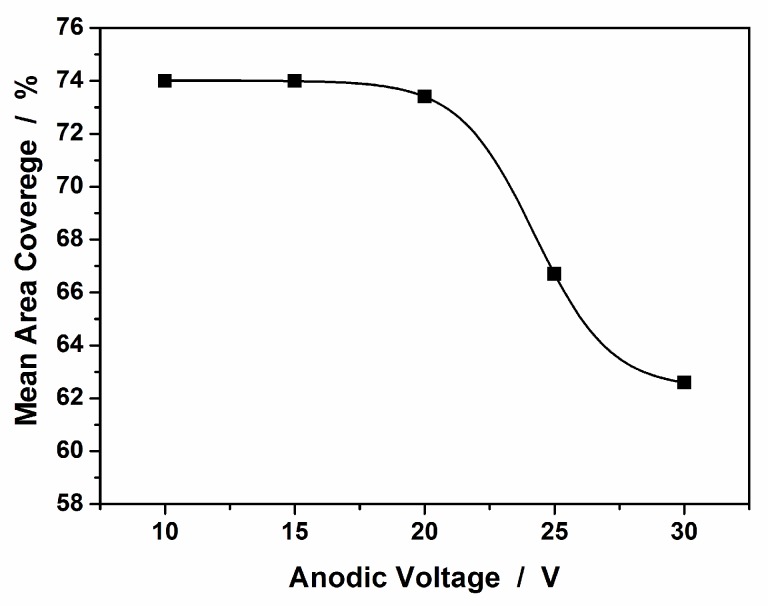
Mean area coverage of TiO_2_ nanotubes anodized on a Ti surface.

Nanotubes grew perpendicular to the titanium substrate and were open at the top and closed at the bottom [46]. The morphology of TiO_2_ nanotubes can be modified relatively easily by controlling the conditions of anodic oxidation, because there was a direct linear relationship between the anodization voltage and the average diameter of the nanotubes formed. In general, the diameter (from 40 nm (10 V) up to 115 nm (30 V)) and wall thickness (from 10 nm (10 V) up to 24 nm (30 V)) of the nanotubes increased with anodic voltage [39], as did the distance of the gaps between the nanotubes. Changing these parameters caused changes in the degree to which the anodized titanium surface was covered by nanotubes (see Figure 5). The average degree of surface coverage for 25 V nanotubes (used in this work) was 67%, which means that 33% of this area was related to free spaces (surface without nanotubes). The results were obtained on the basis of image analysis (ImageJ software [47]) of SEM images registered at the same magnification, and more details are given here [48].

**Figure 6:**
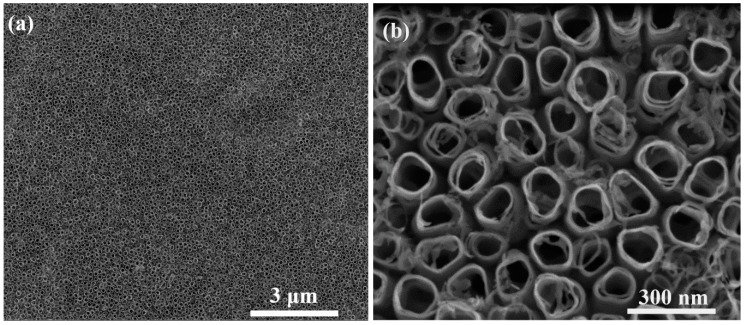
Top view of TiO_2_ nanotubes after anodic oxidation procedure at 25 V in a glycerol-water mixture with ammonium fluoride electrolyte at low (**a**) and high magnification (**b**).

Figure 6a,b show SEM images of the TiO_2_ nanotubes (TiO_2_ NT) after the anodic oxidation procedure at 25 V. The internal diameter of the tubes was about 110 nm, and the wall thickness was about 20 nm [39]. Nanotubes obtained at this voltage were selected for further SERS research. It was related to the fact, that the silver nanoparticles with average size 45 nm after wet deposition were located not only in the spaces between the tubes but also inside them. Therefore, the size of these empty areas (tubes and space between them) can be adjusted to the amount of the Ag cubic nanoparticles. 

Usually, such nanostructures obtained by anodic oxidation are found to be amorphous. Therefore, the TiO_2_ nanotubes were annealed in air at 450 °C in order to change their amorphous structure to a crystalline phase—anatase [39,49], which also led to a transformation of the nanoporous surface towards a hydrophilic surface, as demonstrated by Roguska et al. [39]. The hydrophilic surface promoted the deposition of nanoparticles from aqueous suspension. In addition to physicochemical changes during annealing, we obtained greater mechanical stability of the nanotubes to the titanium substrate by forming the TiO_2_/Ti barrier layer [49]. After heat treatment, the geometry of the tubes did not change. 

**Figure 7:**
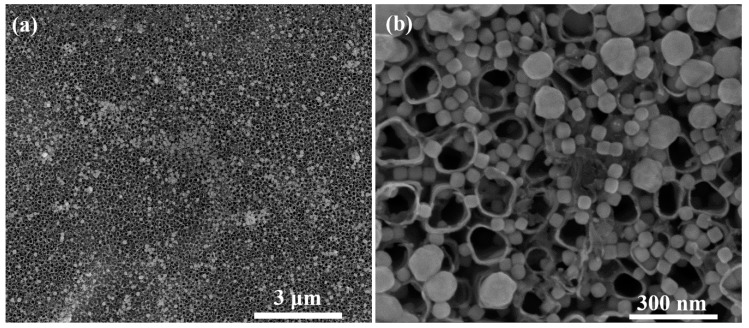
SEM image of Ag cubic nanoparticles deposited by droplet method on titanium oxide nanotubes at low (**a**) and high magnification (**b**).

Subsequently, cubic silver nanoparticles (AgCNPs) were embedded from an aqueous suspension onto annealed TiO_2_ NT substrates. Figure 7a,b show SEM images of the resulting hybrid materials—as can be seen in these figures, the nanoparticles were usually deposited in the slits between individual nanotubes or inside the nanotubes. A specific distribution of the AgCNPs may significantly contribute to the enhancement of the Raman signal through the creation of a significant number of SERS hot spots. In the colloid of nanoparticles used for the preparation of the SERS platform there were a small number of spherical agglomerates that could not be removed during the purification process (see Section 2.2). Therefore, large spherical agglomerates will make some contribution to the measured SERS signal [36,50,51]. According to our earlier studies and literature data, we knew that the SERS activity of layers formed from nanoparticles with sharp edges were significantly larger than the SERS activity of the layer formed from spherical nanoparticles [52,53]. Rycenga et al. showed that the SERS enhancement factor generated by silver nanocubes is about three orders of magnitude larger (×10^3^) than the SERS enhancement factor generated by silver nanospheres with a similar size (measurements were carried out for nanocubes of 38 nm in edge length and nanospheres of 35 nm in diameter) [53]. The increase in the diameter of the nanoparticle may also causes intensification in the generated SERS enhancement factor, however, the observed increase is significantly smaller. For example, Sugawa et al. have shown that the increase in the size of Au/Ag nanoparticles by a factor of 2.4 (from 38 to 90 nm)—similar ratio of the sizes of “big” and “small” nanoparticles were observed in our case (see Figure 7)—led to an increase in the SERS enhancement factor by a factor of 6–8 [54]. This means the contribution from the spherical nanoparticles (even larger and when the distances between them are small, below 10 nm) should be significantly smaller than the contribution from the anisotropic nanoparticles. Therefore, one can assume that the SERS signal will be generated mainly by cubic nanoparticles, where the enhanced E-fields are mainly localized at the corners of the nanocubes. What is also important here is that the cubic nanoparticles were small enough to enter the nanotubes and into the spaces between them, but the spherical agglomerates were too big for it. 

### 3.2. XPS and AES Measurements

**Figure 8:**
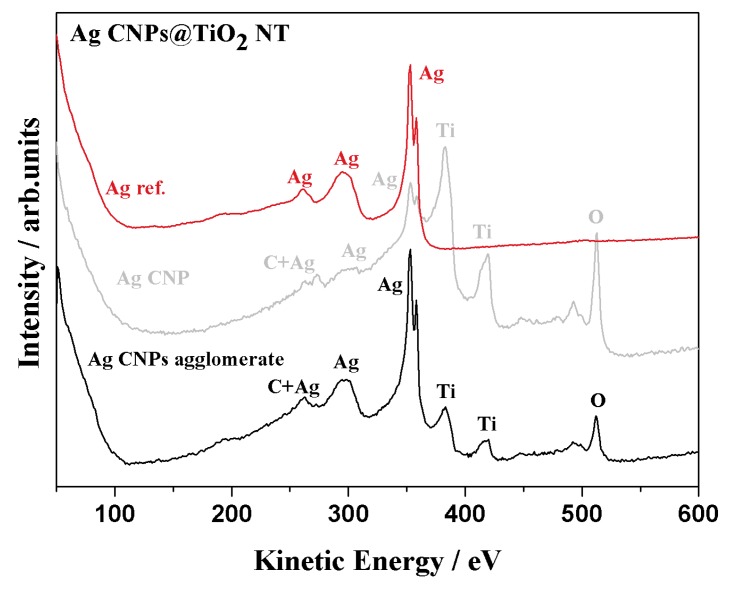
Auger local spectra taken for identification of the chemical composition from a single Ag nanoparticle (gray) and from an Ag agglomerate (black). For comparison, an Ag MNN reference spectrum for metallic Ag is given (red).

Qualitative analyses of the chemical composition of the obtained SERS substrates were performed using Auger spectroscopy. The local Auger spectra recorded with a lateral resolution of a few dozen nm, which depended on the diameter of electron beam (see Figure 8). The Auger signals of the deposited AgCNPs onto TiO_2_ NT support were clearly distinguishable: Ag (MNN), Ti (LMM), O (KLL) and C (KLL). The local Auger spectra, taken from single Ag cubic nanoparticles and from Ag spherical agglomerates, revealed the concentration of these element changes. Such visible differences in Ag concentration can contribute to the formation of SERS active centers, which was also suggested by SEM observations (see Figure 7). 

For determination of surface chemistry of the received SERS platforms, the XPS investigations were performed for titanium oxide nanotubes annealed at 450 °C and at the same substrate with embedded AgCNPs. The measured binding energies of the core electrons provide large amount information about the properties of the atoms in the molecules and solids. Accurate measurements of the XPS peak positions provided a lot more detail about the chemistry of the analyzed atoms than was provided by AES spectroscopy. 

**Figure 9:**
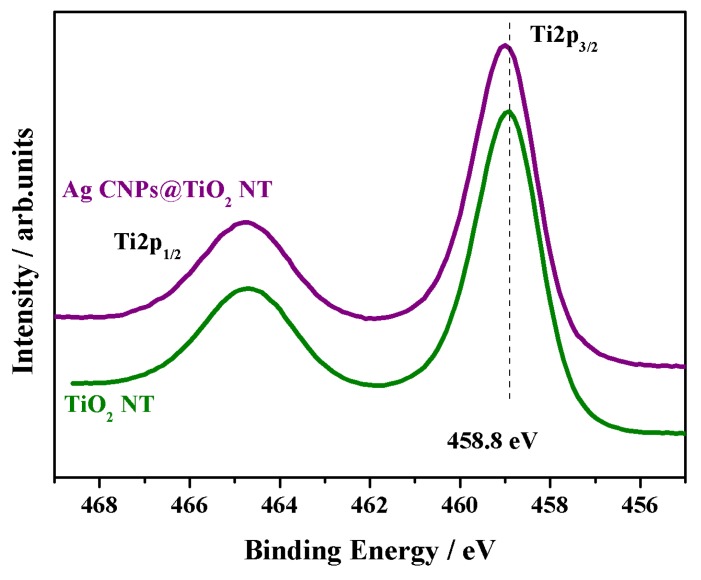
High-resolution X-ray photoelectron spectroscopy (XPS) spectra of Ti2p peaks recorded on titanium dioxide nanotubes annealed at 450 °C (green) and synthesized SERS substrate (violet).

The XPS spectrum of Ti2p for the annealed NTs at 450 °C exhibited two characteristic peaks, which corresponded to Ti2p_3/2_ at 458.8 eV and Ti2p_1/2_ at 464.3 eV, indicating that Ti exists in the form of Ti^4+^. The separation between the two peaks was 5.54 ± 0.2 eV, which was in agreement with XPS standard data [55] (see Figure 9). A small positive shift equal to +0.2 eV was observed in the Ti2p binding energy (BE) for the sample with the AgCNP deposits. The shifted energy (459.0 eV) was also characteristic for titanium dioxide [55]. 

**Figure 10:**
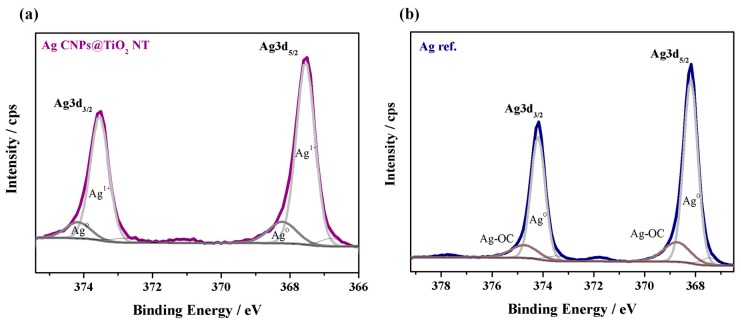
The Ag3d XPS spectra after the deconvolution procedure for AgCNPs deposited on titanium oxide nanotubes (TiO_2_ NT) (AgCNPs@TiO_2_ NT) (**a**) and electrochemically activated silver (Ag reference) (**b**).

Figure 10 presents XPS spectra in the region of the silver Ag3d peaks for the AgCNPs@TiO_2_ NT and the Ag reference sample after the deconvolution procedure, where the separation energy between the Ag3d_5/2_ and Ag3d_3/2_ spectra lines was 6.0 ± 0.2 eV, keeping the ratio of those signals as 3:2, respectively. As can be seen, the binding energy of the Ag3d_5/2_ of the AgCNPs on TiO_2_ NT (367.6 eV) shifted by −0.6 eV relative to 368.2 eV for the metallic silver (electrochemically nanostructured Ag—reference sample). The direction and magnitude of the Ag3d peak shift corresponded to the formation of a thin layer of silver oxide (Ag^1+^) on top of the nanoparticle surfaces, which was consistent with the observations of other authors [56,57]. This was due to the fact that cubic silver nanoparticles were coated with cross-linked PVP polymer (~5 nm thick film), which was confirmed by the STEM microscopic observations (see Figure 4). The second spin-orbit pair of small intensity at higher binding energy values (Ag3d_5/2_ at 368.7 eV and 368.2 eV) was attributed to the oxidized Ag surface bonded with C–O species for the reference sample and metallic silver for the AgCNPs deposited on TiO_2_ NT, respectively. However, some literature data suggested that metallic silver was also possible in an analogous system. Kang et al. and Prieto et al. found silver at ~368.3 [58] and 368.1 eV [59], where the average diameter of Ag nanoparticles was 35 nm. However, our XPS results clearly indicate the presence of PVP on the surface of the cubic nanoparticles, which manifested itself by signals of the characteristic functional groups for this kind of polymer, such as: C=O, C–N and C–C [60]. This was due to the fact that the average inelastic mean free path (IMFP) of an electron in metallic silver is around 1.5–2.0 nm [61,62], therefore, the XPS spectra collected came only from the surfaces of the nanoparticles. In the case of a silver electrode, there was no organic layer that would reduce the depth of the beam penetration. This is why the XPS spectrum showed mainly metal, while silver oxide had only a small share in the spectrum. Thus, the presence of PVP polymer revealed the interfacial interaction between Ag NPs and the PVP molecules.

### 3.3. Raman Measurements

**Figure 11:**
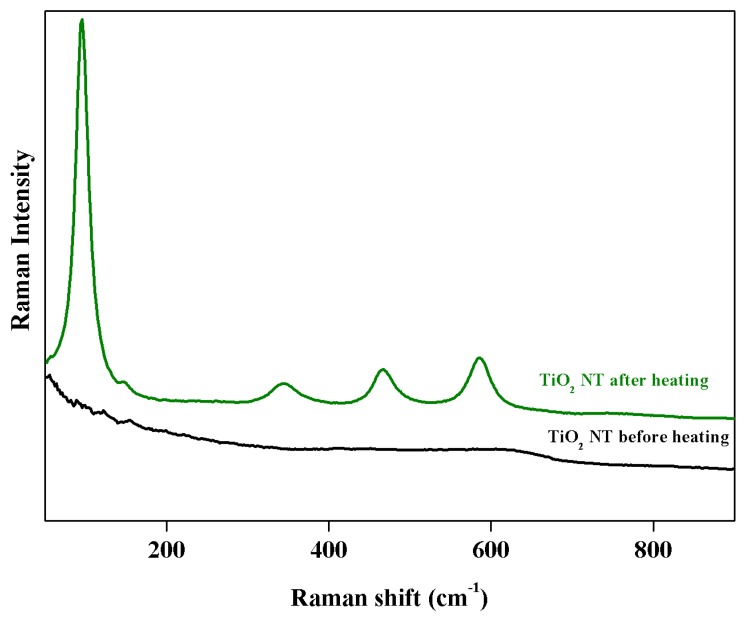
Raman spectra for TiO_2_ NT before (black) and after (green) heat treatment at 450 °C in air (2 h).

It is worth mentioning that it was also possible to observe the transition from amorphous titanium oxide to anatase using Raman spectroscopy. Figure 11 shows the Raman spectra of TiO_2_ NT samples before and after heat treatment at 450 °C for 2 h in air. The crystallographic structure of the nanotubes changed during annealing from the amorphous phase, which was very weak in terms of Raman scattering [63], to the anatase phase. The peaks that appeared were assigned to the vibrations in this phase [64]. 

**Figure 12:**
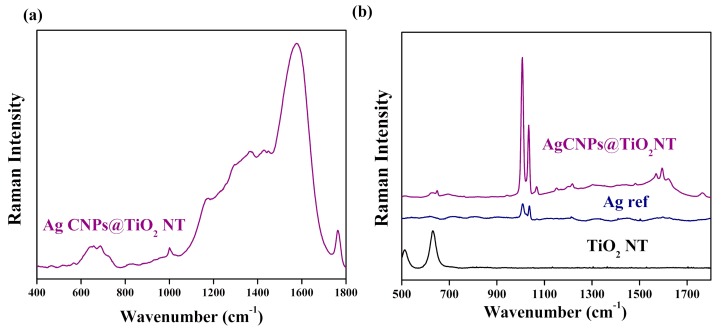
SERS spectra for Ag CNP_S_ deposited on TiO_2_ nanotubes (violet) without pyridine (**a**) and with pyridine (**b**); the SERS reference spectrum for electrochemically activated Ag is also given (blue).

Following this, the Raman spectra of the samples of TiO_2_ NT covered with AgCNPs were recorded (see Figure 12a). They showed Raman bands typical for the carbon clusters produced during the carbonization of PVP (especially in the laser beam) [65,66]. The presence of carbon on the analyzed surface was also confirmed by XPS analysis. In these XPS measurements, a characteristic pyrolidone N group (C–N: 400.2 eV) and adequate bonds for PVP: C–C (285.0 eV), C–O/C=O (286.5 eV, 288.5 eV), C–N (286.5 eV) were detected [60]. 

The spectra recorded for substrates soaked in the pyridine solution (see Figure 12b) showed a difference in the peak gains between the Ag nanoparticles deposited on the TiO_2_ NT substrate and those on the Ag reference platform. No peaks from pyridine were registered on TiO_2_ NT suggesting that this substrate alone did not enhance the pyridine spectrum. The most intense pyridine bands at 1004 and 1034 cm^−1^ originated from the symmetric ring deformation modes (ν_1_) and (ν_12_), respectively [67]. Moreover, SERS spectrum measured using cubic silver nanoparticles deposited on TiO_2_ NTs was characterized by other vibrational bands at ~650 cm^−1^ (ν_6a_), ~1220 cm^−1^ (ν_9a_), ~1600 cm^−1^ (ν_8a_), which originated from vibrations of the aromatic ring of pyridine having A1 symmetry [33,67]. Careful inspection of the obtained SERS spectra revealed also, that the two strongest bands had almost identical intensities for the electrochemically activated surface of silver, which agreed with Zuo and Jogodzinski’s work [67]. A change in the intensity ratio of the breathing doublet mode of pyridine (1004 cm^−1^/1034 cm^−1^) for AgCNPs was observed. This was probably related to the fact that AgCNPs were deposited on nanoporous substrates with specific surface development. Therefore, the interaction between silver nanoparticles coated by PVP and titanium oxide contributed to this behavior (changes near the energy of Fermi level). These factors probably influenced a configuration change in the adsorbate (pyridine) depending on the surface chemistry, morphology of the substrates and active size of silver clusters [68,69].

The enhancement coefficient of the SERS spectra for the Ag CNPs@TiO_2_ NT sample was eight times greater than for the Ag reference sample. From our earlier works, we know that the determined enhancement factor for the reference silver electrode was on the level 4.6 · 10^5^ (the most intense pyridine band—ν_1_). Thus, the resulting substrate had an enhancement factor of about 3.7 · 10^6^ [33,70]. This enhancement was also more than 10 times greater than the nanotubes of the same diameter (25 V—110 nm) coated with spherical nanoparticles (E_F_ = 1.8 ∙ 10^5^), taking into account our previous research [33,70]. The larger gain in the SERS spectrum measured on the cubic Ag nanoparticles deposited on the nanotubular TiO_2_ substrates was probably caused by at least two factors: morphological and chemical. First of all, a careful analysis of the SEM data suggested that the Ag cubic nanoparticles (45 ± 2 nm) were distributed relatively homogenously over the nanoporous oxide surface. Moreover, Ag nanoparticles formed three-dimensional agglomerates on TiO_2_ NT, as seen in the SEM image presented in Figure 7, where additional SERS hot spots may have been formed (a weaker SERS effect in relation to Ag CNPs). SEM observations revealed also that the population of cubic silver nanoparticles was much larger than that of spherical agglomerates. Such morphological changes were not observed for the silver surface after electrochemical activation. 

**Figure 13:**
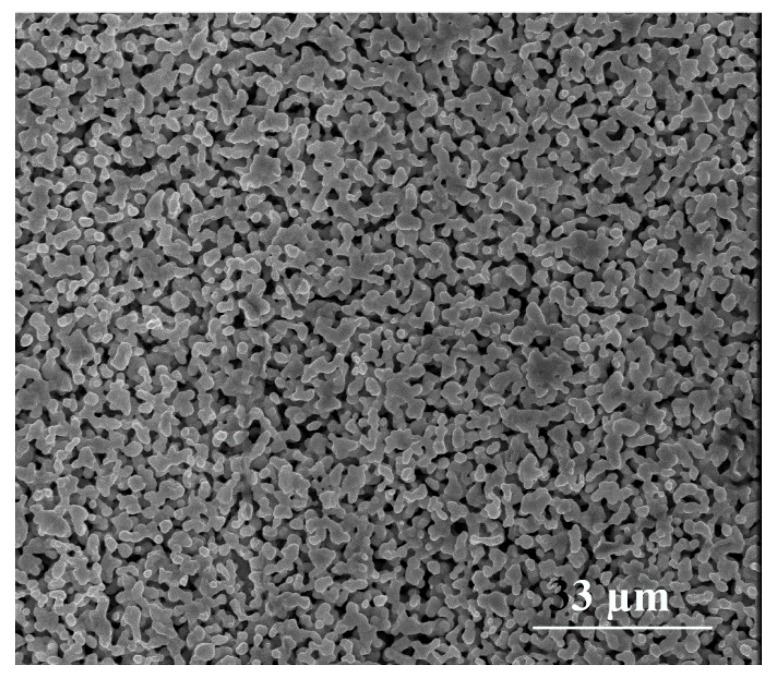
Surface morphology of the silver reference sample.

Figure 13 shows the surface morphology of the pure Ag reference sample after a common roughening procedure: Oxidation–reduction cycling (ORC), which led to a morphology having a large specific surface area. Apparently, a more developed surface area of Ag CNPs deposited on the TiO_2_ NT offered a larger population of active SERS sites, such as slits or narrow cavities. This meant that the higher SERS signal enhancement was a result of the way the Ag deposit distributes itself on the nanotubular substrate.

Secondly, interactions between the nanocubes and nanotubes may increase the final enhancement of the SERS spectrum through what is known as the chemical mechanism—CT (charge transfer) effect [71,72,73]. The CT process is considered to be a resonant Raman-like enhancement associated with the excited state of the molecule–metal system and the charge transfer between the molecule and the metal surface [74]. Therefore, the use of semiconductors such as TiO_2_ nanotubes may result in an additional spectral amplification due to a charge transfer effect between the semiconductor, metal nanoparticles and adsorbents [71,72,73] and, moreover, between the PVP molecules and metal noble nanoparticles, as was observed by other authors [60]. This phenomenon is usually associated with a reduction in the electron density of the pyrolidone group as a result of a charge-transfer-type interaction between the PVP molecules and surface of the AgCNPs. The sum of these interactions was visible in the XPS titanium spectra, where a shift in the Ti2p_3/2_ peak of about 0.2 eV was observed for the sample with the silver deposit on the TiO_2_ NT with respect to the uncoated sample (see Figure 9). 

**Figure 14:**
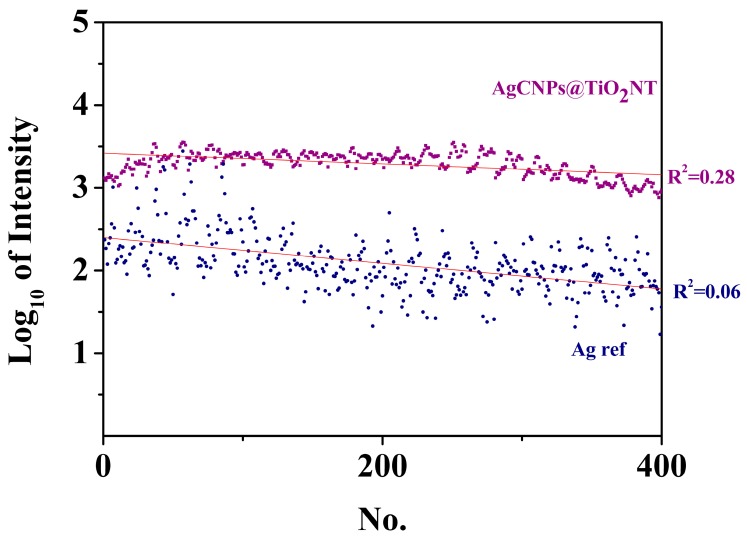
Logarithmic plot of the intensity of the pyridine band at 1004 cm^−1^ recorded in each of the series of 400 measurements for pyridine adsorbed at AgCNPs@TiO_2_ NT (violet) and silver electrode (blue).

We also found that the reproducibility and temporal stability of the SERS signals intensities was better for the nanoporous titanium oxides substrates with silver deposit than for the standard roughened Ag, which is an important factor for potential applications of such materials. Figure 13 shows that the difference in the signal intensity at 1004 cm^−1^ for subsequent measurements at a different sample site (offset by 2 microms) was significantly lower for the hybrid layer produced than for the silver nanostructured electrode. In addition, it can be seen that it was possible to register spectra with a similar peak intensity on the Ag reference sampe, but only in characteristic places with the largest amplification parameter. This was not necessarily the case with the prepared substrate, which showed its greater utility in analytical applications. Moreover, as can be seen in Figure 14, for both substrates, the well-known process of a temporal decrease in their SERS activity was observed [57,66]. This deactivation process (usually activated photochemically or by the application of very negative potential) was mainly caused by the incorporation within the bulk metallic phase of very small silver clusters, which were especially active in enhancing the SERS signal by the CT mechanism [57,66]. The deactivation process was significantly more important for the standard SERS substrates (Ag reference) than for the AgCNPs@TiO_2_ NT hybrid material (see Figure 14). Signal oscillations visible in Figure 14 resulted probably from the measurement method. The spectra were collected from a square area of 40,000 µm, where subsequent measuring points were made from left to right. There were 20 measurement points in one row, which corresponded to the frequency of oscillations shown in Figure 14. In areas with increased peak intensities, larger agglomerates of nanoparticles were likely to be found.

**Figure 15:**
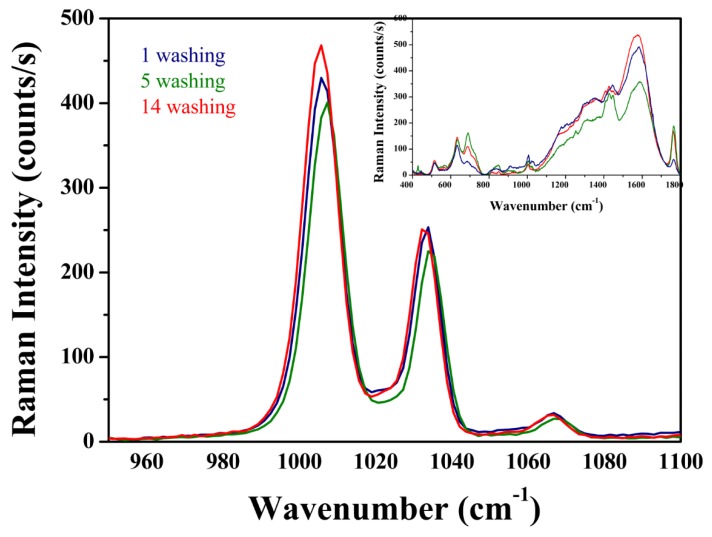
SERS spectra for Ag CNP_S_ deposited on TiO_2_ nanotubes with pyridine and without pyridine (insert) after 14 cycles of water washing.

An important factor in the design of active SERS platforms was also the possibility of utilizing them for multiple use. Therefore, Figure 15 shows the change in the intensity of the SERS pyridine signal—a characteristic doublet (breathing mode) in relation to the number of washing cycles of the platform surface (AgCNPs@TiO_2_ NT) by water. It is clearly visible that the intensity of the measured doublet was practically unchanged. This means that the prepared SERS substrates retained the enhancement parameter at a similar level and had morphological stability during subsequent water washings. Cubic silver nanoparticles were well-placed in the slits between nanotubes and inside of the nanopores, as can be seen in Figure 7. The Figure 15 insert presents also the SERS spectra recorded on the surface of the AgCNPs@TiO_2_ NT immediately after water washing, without pyridine. It can be observed that after the removal of pyridine, the surface chemistry of the prepared SERS substrate did not change. Only a characteristic spectrum in the initial state is clearly visible, as in Figure 12a.

## 4. Conclusions

Stable, reproducible and highly active platforms for SERS investigations were obtained using relatively simple electrochemical and chemical procedures. The SERS platform based on TiO_2_ nanotubes and cubic silver nanoparticles thus obtained increased the SERS signal by a factor of eight as compared with the standard electrochemically nanostructured Ag surface (SERS substrate). Such large enhancement of the SERS signal was a result of specific morphology and surface chemistry of TiO_2_ nanotubes which fixed on them, Ag cubic nanoparticles. The strong LSPR effect was localized in a short distance between nanoparticles, where the sharp corners played a crucial role. Stability for the received SERS platform in relation to Ag reference sample was also better. It was associated with a distribution of silver nanoparticles on the nanotubes surface, so that there was a significantly greater amount of SERS active places, or “hot spots”. For silver nanoparticles deposited on the TiO_2_ substrate, a lower deactivation process of the active surface was also observed. Prepared platforms at nanoscale were stable, even after washing them several times by water. This means that they can be used many times in SERS measurements (using the same analyte). All these factors make titanium oxide nanotubes with embedded cubic silver nanoparticles attractive substrates for SERS applications.
